# Commentary: Novel Insight into the Genetic Basis of High Altitude Pulmonary Hypertension in Kyrgyz Highlanders

**Published:** 2019-03-19

**Authors:** Tsering Stobdan, Gabriel G. Haddad

**Affiliations:** 1 Division of Respiratory Medicine, Department of Pediatrics, University of California, San Diego, La Jolla, CA 92093, USA; 2 Department of Neurosciences, University of California, San Diego, La Jolla, CA 92093, USA; 3 Rady Children’s Hospital, San Diego, CA 92123, USA

Human populations living at high altitude (HA) is an experimental setup to study gene-environment interactions^1–3^. The fact that acute HA exposure is associated with illnesses like cerebral and pulmonary edema, which when untreated are lethal, provides a clear notion that evolutionary selection to high altitude hypoxic stress had been indispensable to their current survival. Such adaptations are well studied in the four well recognized HA populations i.e., Ethiopian (Africa), Tibetan and Kyrgyz (Asia) and Andean (South America)^4, 5^. For example, the prevalence of high altitude pulmonary hypertension (HAPH) is 14–20% in the Kyrgyz population^5^ the remaining being resistant or adapted. Since hypoxia is the common factor in many disease pathophysiologies, the candidate genes identified to have a role in HA adaptation in these populations have direct implications for therapeutic purposes.

In the recent study entitled ‘Novel insight into the genetic basis of High Altitude Pulmonary Hypertension in Kyrgyz highlanders’,^6^ we analyzed the first whole genome sequence of Kyrgyz highlanders to identify genomic intervals selected for HAPH. It is important to note that HAPH was first described in the Andean population^7^, however this population also shows other potentially confounding characteristics such as polycythemia, which, in turn, can lead to HAPH. The Kyrgyz population on the other hand provides a unique opportunity to study HAPH because they do not present any other features related to chronic hypoxia besides HAPH^5^. In order to capture all the possible candidate DNA-selected intervals we performed whole genome sequencing of the case-control cohort at a higher resolution of 40x. The cohort itself includes a very well defined HAPH patients and healthy controls recruited from a tertiary level of screening^8^ further complemented our study. The robust inclusion and exclusion criteria included measurements from right heart catheterization, even in the case of healthy controls.

Our results have revealed out some very interesting candidate genes which likely lead to HAPH in this population. This includes genes *MTMR4*, *TMOD3* and *VCAM1* that are functionally associated with well-known molecular and pathophysiological processes for pulmonary hypertension (PH). Such processes are as follows: i) dysfunctional BMP-signaling, ii) disrupted tissue repair processes and iii) abnormal endothelial cell function. Simultaneously, through enhanced sequence resolution, we could also analyze the mutations within the 33 candidate genes linked to HAPH in the same population by whole exome analysis^9^. Interestingly, only one gene i.e., *SIGLEC11*, out of these 33 candidates was in the top candidate interval in our current study, the difference likely being due to the difference between whole genome and exome sequencing.

It is important to highlight that clinically HAPH is categorized to Group-3 PH i.e., PH due to lung disease or hypoxia (or both)10. This group also includes chronic obstructive pulmonary disease (COPD), interstitial lung disease and sleep apnea. The other groups includes idiopathic, familial/heritable, to presenting as a secondary disease e.g., congenital heart disease with left-to-right shunt^10^. Within Group-3 PH, the prevalence of COPD alone is >300 million people and is ranked as the third most leading cause of death globally^11^. Similarly, recent report suggest that sleep apnea in the general adult population ranged from 6% to 17%, being as high as 49% in the advanced ages^12^. As we know that the prevalence of PH in both COPD and sleep apnea increases with disease severity, the understanding of the molecular basis of PH would be critical especially for the maximum risk patients. Since hypoxemia, chronic hypoxemia in COPD and intermittent in sleep apnea, is the conjoining factor that relates these diseases with HAPH, the candidate genes that we have identified for HAPH from this unique population will have implications in understanding the molecular basis of disease progression in the lungs. Additionally, because these genes are novel and are not yet studied for its role in other forms of PH, they can be prospectively evaluated as candidates in future studies on PH in general. For example pulmonary arterial hypertension (PAH), which is between 6 and 15 cases per million adults^13, 14^ and adding ~1000 patients each year in the United States alone^15^ includes heritable form of PH where mutations in the bone morphogenetic protein receptor type 2 (*BMPR2*) gene is one of the well know candidate. Interestingly, the candidate gene *MTMR4* that we identified in our recent study is downstream to the BMP receptors and may be a proxy to *BMPR2* mutations in the Kyrgyz HAPH cases. Similarly, in idiopathic PH, which is a progressive disorder that does not depict any underlying cardiopulmonary or other medical conditions but culminates in right ventricular failure and death, the role of these genes can be evaluated for their characterization. Therefore, the current study not only provides an opportunity to understand the biology of susceptibility to HAPH in HA population, but will also provide novel genes to further study in other forms of PH or more precisely to the Group-3 pulmonary hypertension. In other words, such effort would not only benefit the native population, for example >14% of Kyrgyz population, but will also benefit other PH patients for whom the available treatments are only to relieve the symptoms and slow the progress of the disease.
